# New Method of Disinfecting and Deodorising

**Published:** 1865-11

**Authors:** 


### New Method of Disinfecting and Deodorising.

At the recent meeting of the British Medical Association, Dr. Richardson explained a process he had adopted for applying the atomiser for the purpose of deodorisation. He made a mixture by adding iodine to solution of peroxide of hydrogen until saturation occurred, and afterwards concentrated sea-salt in proportion of 2½ per cent. In this combination a water was produced like sea-water, and which was rendered active by being charged with free iodine and ozone. The solution placed in one of Krohne's hand antomisers could be diffused in the finest state of distribution at the rate of two fluid ounces in a quarter of an hour; but in an ordinary bed-room or sitting-room one ounce was sufficient to render the air so active that ozone test-papers were discolored by it to the highest degree of Moffat's scale in from five to ten minutes. For charging the sick room rapidly and effectually with active air—in a word, with sea-air—Dr. Richardson said this plan was by far the most effective of any he had known. A nurse could put the apparatus into action at once, and could deodorize hour by hour, according to the directions of the medical practitioner.—*Druggist.*

